# The preventive effect of Qing Dai on bisphosphonate-induced gastric cellular injuries

**DOI:** 10.3164/jcbn.17-108

**Published:** 2018-11-15

**Authors:** Go Yasuda, Hiromu Ito, Hiromi Kurokawa, Masahiko Terasaki, Hideo Suzuki, Yuji Mizokami, Hirofumi Matsui

**Affiliations:** 1 Faculty of Medicine, University of Tsukuba, 1-1-1 Tennodai, Tsukuba, Ibaraki 305-8575, Japan; 2 Graduate School of Medical and Dental Sciences, Kagoshima University, 8-35-1 Sakuragaoka, Kagoshima 890-8544, Japan; 3 Kyoto Prefectural University of Medicine, 465 Kajii-cho, Kawaramachi-Hirokoji, Kamigyo-ku, Kyoto 602-8566, Japan

**Keywords:** Qing Dai, herbal medicine, reactive oxygen species, mitochondria, bisphosphonate, gastrointestinal injury

## Abstract

The Chinese herbal medicine Qing Dai has been traditionally used for the treatment of various inflammatory diseases. We previously reported that reactive oxygen species play an important role in bisphosphonate-induced gastrointestinal injuries and that Qing Dai improved ulcerative colitis by scavenging reactive oxygen species. In this study, we investigated whether Qing Dai prevented bisphosphonate-induced gastric cellular injuries. Risedronate (a bisphosphonate) was added to rat gastric mucosal cells. Risedronate-induced cellular injury, cellular lipid peroxidation, mitochondrial membrane potential, and reactive oxygen species production in rat gastric mucosal cells were examined via viable cell counting, specific fluorescent indicators, and electron spin resonance. Pretreatment with Qing Dai attenuated the fluorescence intensity of diphenyl-1-pyrenylphosphine and MitoSox as well as the signal intensities of electron spin resonance. Cell viability improved from 20% to 80% by pretreatment with Qing Dai. Thus, Qing Dai prevented this injury by suppressing mitochondrial reactive oxygen species production, which is the main cause of cellular lipid peroxidation. Qing Dai also maintained mitochondrial potential, reducing reactive oxygen species production. We conclude that Qing Dai has protective effects on bisphosphonate-induced gastrointestinal injury and thus has the potential for clinical application.

## Introduction

Qing Dai (QD), also known as Indigo naturalis, is a component of Chinese herbal medicines. QD is a navy powder extracted from the leaves and roots of plants such as *Strobilanthes cusia* and *Isatis tinctoria*.^(1)^ QD consists of indigo, indirubin, isoindigotin, tryptanthrin, and nimbosterol.^(1,2)^ Lin Y *et al.*^(3)^ reported that the extract of QD had the ability to scavenge reactive oxygen species (ROS) and suppressed inflammation. QD has been used for treatment of recalcitrant psoriasis, mouth ulcers, and radiation proctitis.^(4–6)^ An *in vitro* study showed that the extract of QD inhibited the proinflammatory response of human neutrophils, while a study of QD *in vivo* showed that the colon, when injured by dextran sodium sulfate (DSS), had significantly milder results when QD was administered.^(2,7)^ We reported that QD restrained inflammation in patients with ulcerative colitis (UC) and attenuated non-steroidal anti-inflammatory drug (NSAID)-induced gastrointestinal epithelial cellular injuries by scavenging ROS.^(1,8)^

Osteoporosis is characterized by the loss of bone mass and strength, often leading to fractures. As the average human lifespan increases, this disease is becoming a major clinical problem.^(9)^ Osteoporosis caused more than 9 million fractures worldwide in 2000.^(10)^ In Japan, there are 15 million patients with osteoporosis and 130,000 patients who suffered from hip fractures.^(11)^ Patients with bone diseases such as glucocorticoid-induced osteoporosis, postmenopausal osteoporosis, and Paget’s disease are advised to take nitrogen-containing bisphosphonates (BP) such as risedronate and alendronate.^(12,13)^ However, oral administration of BP may result in gastrointestinal side effects such as gastric ulcer, erosive esophagitis, and gastritis.^(14)^ *In vivo* studies have shown that BP acts as a topical irritant to induce gastrointestinal injury without decreasing prostaglandin synthesis.^(12,14,15)^ We have recently reported that BP induces mitochondrial dysfunction and subsequent generation of ROS such as superoxide (O_2_^•−^).^(16)^ ROS induces cellular lipid peroxidation, which can lead to cell death. This mechanism is related to BP-induced gastrointestinal injury.^(16)^ Therefore, a reagent that scavenges ROS may help reduce the harmful effects of BP on gastrointestinal tracts.

In this study, we assessed whether QD can reduce BP-induced gastric epithelial cellular injury. We used a gastric mucosal cell line, RGM1, to investigate the preventive effects of QD on BP-induced ROS by assessing electron spin resonance spectroscopy (ESR), cytotoxicity, cellular lipid peroxidation, and the influence on mitochondrial membrane potential.

## Materials and Methods

### Materials

Risedronate was obtained from LKT Laboratories, Inc. (St. Paul, MN). QD powder was purchased from Seishinshouakudo (Tokyo, Japan), which imports QD from China. Cell Counting Kit-8 (CCK-8), diphenyl-1-pyrenylphosphine (DPPP), and MitoRed were purchased from Dojindo (Kumamoto, Japan). 5,5-Dimethyl-1-pyrroline-*N*-oxide (DMPO) was purchased from LABOTEC Co., Ltd. (Tokyo, Japan) and β-nicotinamide adenine dinucleotide (NADH), d-glutamic acid, and succinic acid were purchased from Sigma-Aldrich (St. Louis, MO). Malic acid, indirubin, and indigo were purchased from Wako Pure Chem. Ind., Ltd. (Osaka, Japan) and MitoTracker Green FM was purchased from Cell Signaling Technology Japan, K.K. (Tokyo, Japan).

### Cell culture

A rat gastric epithelial cell line (RGM1) established by our research group was used.^(17)^ Cells were grown in a 1:1 mixture of Dulbecco’s Modified Eagle’s Medium (DMEM) and Ham’s F-12 medium (Life Technologies Co., Carlsbad, CA), supplemented with inactivated 10% fetal bovine serum (FBS; Biowest LLC, Kansas City, MS) and 1% penicillin and streptomycin (Life Technologies Co.). The cells were cultured at 37°C in a humidified incubator with 5% CO_2_.

### Cell viability test

Cell viability was evaluated using CCK-8 according to the manufacturer’s instructions. Briefly, cells were seeded on a 96-well polypropylene plate at a concentration of 1 × 10^4^ cells/well. After overnight incubation, the medium was replaced with fresh medium containing 0, 1.25, 2.5, or 5 µg/ml QD and cells were further incubated for 2.5 h. Cells were washed with phosphate-buffered saline (PBS) twice, and fresh medium containing 100 µM risedronate was added to each well. After 24 h incubation, the medium was replaced with fresh medium containing 10% (v/v) CCK-8. The absorbance of each well was measured at 450 nm with a multimode plate reader (DTX 880; Beckman Coulter, Fullerton, CA). Pretreatment and treatment’s time is according to the previous studies.^(8,16)^

### Measurement of cellular lipid peroxidation

DPPP is a non-fluorescent triphenylphosphine compound that reacts with hydroperoxide to form the fluorescent compound DPPP-oxide. After pretreatment with or without 5 µg/ml QD for 2.5 h, cells were treated with 100 µM risedronate for 24 h, followed by 5 µM DPPP for 15 min. The cells were then washed with PBS twice and resuspended in fresh medium. Fluorescence images of cells were obtained with a chilled CCD camera mounted on an epifluorescence microscope (BZ-X710, KEYENCE, Osaka, Japan) with the excitation (ex.) and emission (em.) wavelengths at 360 and 405 nm, respectively. The fluorescence intensities were analyzed using ImageJ 1.50i.

### Electron spin resonance spectroscopy

ROS generation in the cells was measured using ESR in accordance with previous studies.^(18,19)^ Briefly, cells were seeded onto a glass cover slide (49 × 5 × 0.2 mm) and incubated overnight. They were incubated with 100 µM risedronate for 24 h with or without 5 µg/ml of QD pretreatment for 2.5 h. They were immersed in respiratory buffer containing 5 mM succinate, 5 mM malate, 5 mM glutamate, 5 mM NADH, and 5 µl DMPO as a spin-trapping agent. The cell-attached glass cover slide was placed on a tissue glass slide, and the ESR spectra were obtained by inserting the slide into the device. All ESR spectra were obtained using a JEOL-TE X-band spectrometer (JEOL Ltd., Tokyo, Japan) under the following conditions: 10 mW incident microwave power, 9.42 GHz frequency, and 0.1 mT field modulation amplitude. Radicals such as O_2_^•−^ and hydroxyl are unstable objects and disappear rapidly at room temperature, making it difficult for ESR to detect radicals directly.^(20)^ Spin-trapping agents such as DMPO can bind to and stabilize radicals. For example, O_2_^•−^ is stabilized by binding with DMPO to become DMPO–OOH. ESR can detect radicals that have been stabilized by a spin-trapping agent.

### Measurement of mitochondrial superoxide

 Superoxide leakage in mitochondria was detected using a fluorescence indicator, MitoSOX, and localization of mitochondria was detected using another fluorescence indicator, MitoTrackerGreen. MitoSOX permeates living cells and accumulates in mitochondria selectively. It is rapidly oxidized by O_2_^•−^ but not by other ROS and reactive nitrogen species (RNS). Cells were treated with 5 µg/ml QD for 2.5 h and then washed with PBS twice before being treated with risedronate 100 µM for 24 h. The cells were incubated for 30 min in 5 µM MitoSOX and 100 nM MitoTrackerGreen diluted with medium. After incubation, the cells were washed with PBS twice. Fluorescence images of MitoSOX (ex. 545 nm, em. 605 nm) and MitoTrackerGreen (ex. 470 nm, em. 525 nm) were captured using an epifluorescence microscope and the fluorescence intensities of MitoSOX were analyzed using ImageJ 1.50i.

### Measurement of cellular mitochondrial transmembrane potential

Cellular mitochondrial membrane potential was detected with a cell-membrane-permeable, rhodamine-based dye, MitoRed. The fluorescence intensity depends on mitochondrial membrane potential; therefore, this dye can be used as an indicator of mitochondrial activity.^(8)^ The cells were treated with 100 µM risedronate with or without 5 µg/ml QD, as mentioned above. The fluorescence images were obtained using an epifluorescence microscope with excitation and emission wavelengths at 545 and 605 nm, respectively. The fluorescence intensities were analyzed using ImageJ 1.50i.

### Statistical analysis

Data are expressed as means ± SD. The statistical significance of the data was evaluated using SPSS Statistics software (ver. 23.0, IBM Corporation, Armonk, NY). Tukey’s test was used for comparison of more than two data sets; *p*<0.05 and *p*<0.01 were considered statistically significant.

## Results

### Qing Dai pretreatment prevented BP-induced cellular injury

**Fig. 1:**
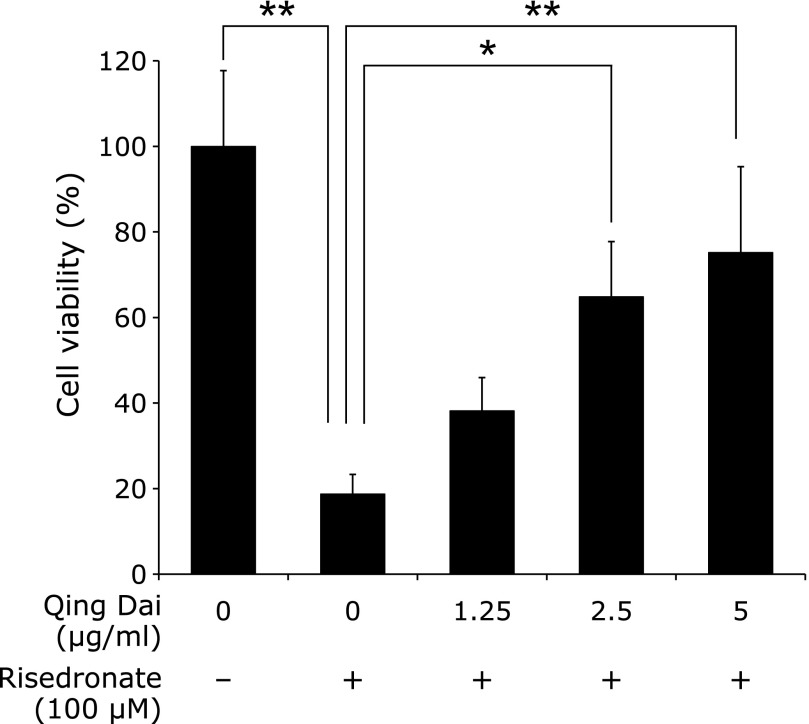
Cell viability was calculated using the CCK-8 colorimetric assay. The prevention amount of QD for risedronate-induced cell injury was estimated. Data are expressed as percentages of untreated cells (mean ± SD). *n* = 4, *p<0.01, **p<0.05, Tukey’s test.

To examine whether QD prevents gastric cellular injury by BP, we pretreated RGM1 cells with QD and incubated them with risedronate. We then examined cellular viability with a CCK-8 colorimetric assay. Risedronate (100 µM) was cytotoxic to RGM1 cells and the viability was approximately 20% compared to the control. However, pretreatment with QD restored cellular viability in a dose-dependent manner to approximately 80% (Fig. 1). This result indicates that QD prevented cytotoxicity caused by risedronate.

### Qing Dai prevented cellular peroxidation induced by BP treatment

**Fig. 2:**
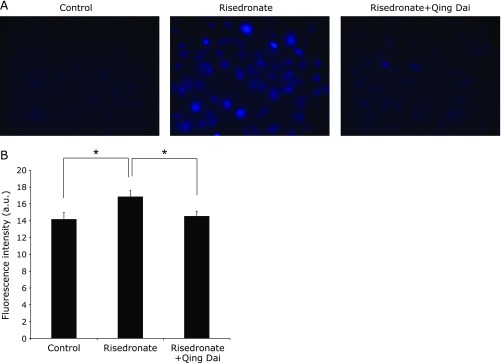
BP-induced cellular lipid peroxidation levels were evaluated by measuring DPPP-oxide fluorescence. RGM1 cells were pretreated with or without 5 µg/ml QD for 2.5 h and treated with 100 µM risedronate for 24 h. A: Fluorescence images were obtained using a chilled CCD camera mounted on an epifluorescence microscope (40×). B: The data shows the fluorescence intensity of DPPP (mean ± SD). The excitation and emission wavelengths are at 360 nm and 405 nm, respectively. *n* = 4, *p<0.05, Tukey’s test.

We analyzed the effect of QD on BP-induced lipid peroxidation by using DPPP. DPPP gains fluorescent properties upon reaction with hydroperoxide. Treatment with risedronate alone showed higher fluorescence intensity, whereas pretreatment with QD attenuated the fluorescence intensity. These results indicated that pretreatment with QD prevented cellular peroxidation induced by risedronate (Fig. 2).

### Reduction of ESR signal by Qing Dai

**Fig. 3:**
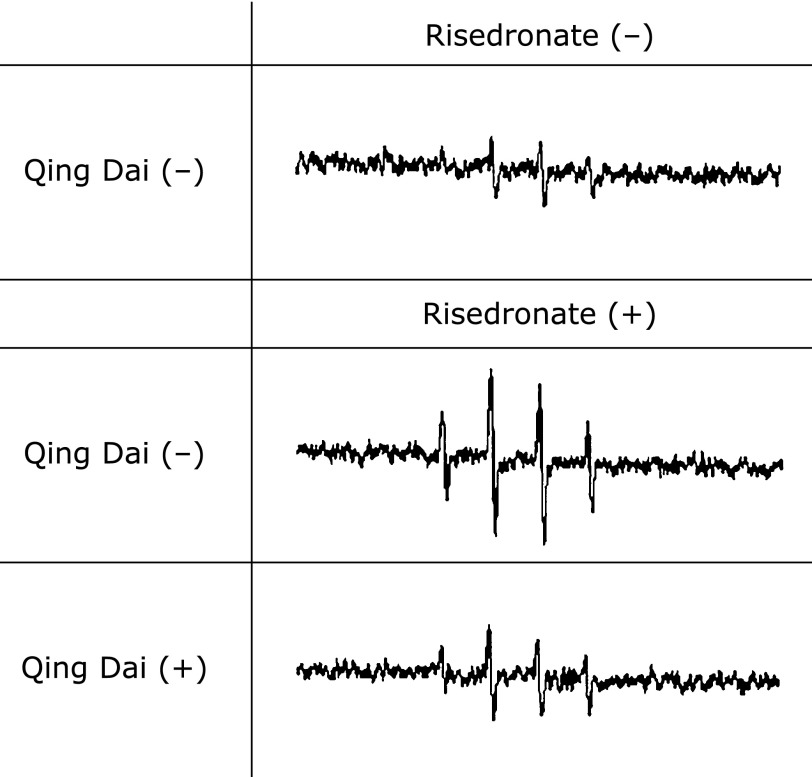
Electron spin resonance (ESR) was used to examine cellular ROS generation. Cells were pretreated with 5 µg/ml QD for 2.5 h and treated with 100 µM risedronate for 24 h. Cells were in a respiration buffer containing a spin trapping agent, DMPO, when the measurements were performed.

We performed ESR analysis on risedronate-treated RGM1 cells, with or without QD pretreatment, using a spin-trapping reagent, DMPO. Figure 3 shows that ESR signals of DMPO–OH were detected. The signal intensity of DMPO–OH reflects the amount of O_2_^•−^ because DMPO–O_2_^•−^ is easily changed to DMPO–OH.^(21)^ The DMPO–OH signal of risedronate-treated cells without QD pretreatment was stronger than that of risedronate-treated cells with QD pretreatment. These results indicate that QD attenuates ROS induced by exposure of risedronate.

### Qing Dai attenuated mitochondrial reactive oxygen production

**Fig. 4:**
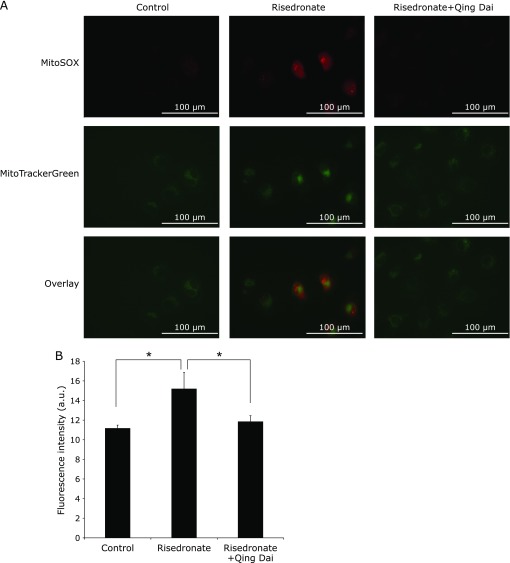
BP-induced superoxide in cells was visualized with MitoSOX fluorescent dye and cellular mitochondria were observed with MitoTrackerGreen fluorescent dye. RGM1 cells were pretreated with or without 5 µg/ml QD for 2.5 h and treated with 100 µM risedronate for 24 h. A: Fluorescence images were obtained with a chilled CCD camera mounted on an epifluorescence microscope (60×). MitoSOX selectively accumulates in mitochondria and immediately reacts with superoxide, resulting in red fluorescence. MitoTrackerGreen enters mitochondria and shows green fluorescence. B: The data shows the fluorescence intensity of MitoSOX (mean ± SD). The excitation and emission wavelengths of MitoSOX are at 545 nm and 605 nm, respectively. The excitation and emission wavelengths of MitoTrackerGreen are at 470 nm and 525 nm, respectively. *n* = 4, *p<0.05, Tukey’s test.

We reported that NSAID induced mitochondrial injury and subsequent overproduction of ROS from mitochondria.^(22)^ We also previously reported that QD decreased the amount of mitochondrial ROS induced by NSAID.^(8)^ In this study, we investigated whether the BP-induced ROS were derived from mitochondria and used microscopic analysis to determine if QD could reduce the ROS. Treatment with risedronate alone showed a significant increase in MitoSOX fluorescence, whereas risedronate-untreated control did not (Fig. 4). In addition, the fluorescence of MitoSOX coincided with the fluorescence of MitoTrackerGreen. These results indicated that risedronate induced the overproduction of mitochondrial ROS. Additionally, the risedronate-induced fluorescence of MitoSOX derived from mitochondrial ROS was highly decreased upon pretreatment with QD. This result indicated that the leakage of O_2_^•−^ from mitochondria induced by risedronate was reduced by QD.

### Qing Dai inhibited the decrease of mitochondrial transmembrane potentials

**Fig. 5:**
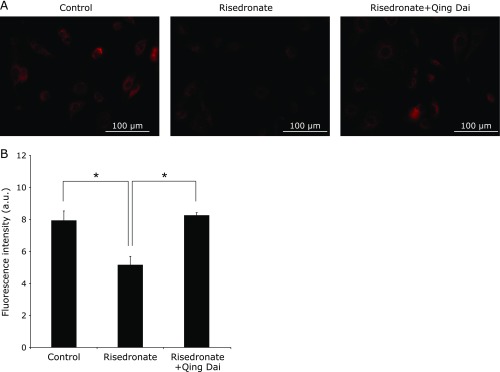
Mitochondrial membrane potential of BP-treated cells was analyzed using MitoRed fluorescent reagent. RGM1 cells were pretreated with or without 5 µg/ml QD for 2.5 h and treated with 100 µM risedronate for 24 h. A: Fluorescence images were obtained with a chilled CCD camera mounted on an epifluorescence microscope (40×). B: The data show the fluorescence intensity of MitoRed (mean ± SD). The excitation and emission wavelengths of MitoRed are at 545 nm and 605 nm, respectively. *n* = 4, *p<0.05, Tukey’s test.

Exposing gastric cells to BP caused a decrease in mitochondrial transmembrane potentials by inducing the formation of ROS.^(16)^ We examined whether QD inhibited the decrease of mitochondrial transmembrane potentials. MitoRed, which is an indicator of mitochondrial transmembrane potentials, was used to examine gastric cellular mitochondrial transmembrane potentials. Examined cells were treated with risedronate and pretreated with or without QD. Whereas cells pretreated without QD showed little MitoRed fluorescence, cells with QD showed strong red fluorescence (Fig. 5). This indicated that QD inhibited the decrease of mitochondrial transmembrane potentials induced by risedronate.

## Discussion

Previous studies have reported that BP induces gastrointestinal injury.^(12,14,15)^ We have clarified the underlying mechanism, which was attributed to a mitochondrial dysfunction due to BP treatment that reduced the mitochondrial membrane potential and increased ROS production levels.^(12,14–16)^ In this study, we demonstrated that QD, a component of Chinese herbal medicine for inflammatory bowel diseases, prevented BP-induced gastric cellular injuries. QD suppressed both BP-induced cellular injury and lipid peroxidation. In addition, QD reduced ROS production and maintained mitochondrial membrane potentials. These results implied that QD could attenuate adverse effects of BP at the molecular and cellular levels.

In previous studies, we demonstrated that mitochondrial membrane potential and cell viability were reduced by exposing gastric intestinal cells to gastric acid and NSAID, BP.^(8,16,23)^ This study also indicated that BP reduced mitochondrial membrane potential. NSAID and gastric acid reducing mitochondrial membrane potential cause mitochondrial permeability transaction and subsequently induce O_2_^•−^ by liberating cytochrome *c* from mitochondrial intermembranous space into cytosol.^(23)^ Previous studies indicated that BP injured mitochondria as well as this study.^(16,24)^ Mitochondria vulnerable to oxidative stress and it was reported that degeneration of mitochondria was associated with Alzheimer’s disease and diabetes.^(25,26)^ O_2_^•−^ induced by mitochondria injury the other mitochondria and amount of O_2_^•−^ increase subsequently. The increase of superoxide from the mitochondrial electron transport chain subsequently accelerates cellular lipid peroxidation and apoptosis in gastric epithelial cells.^(16)^ However, the biologic mechanism that BP injures gastric cells’ mitochondria is unknown.^(16)^

Furthermore, we considered another mechanism of QD and examined whether QD inhibited cellular uptake of BP by measuring absorbance of BP. However, the difference between the absorbance of BP-treated cells and the absorbance of both QD and BP-treated cells were not detected (Supplemental Fig. 1*). Thus, QD does not inhibit intracellular uptake of BP. So, this study indicated that QD protected mitochondria which were damaged by BP. The mechanism of cytoprotection by QD is likely related to both directly scavenging ROS and protecting mitochondria. NSAID inhibit oxidative phosphorylation to reduce the mitochondrial transmembrane potential, which leads to an increase in ROS and mitochondrial injury. BP may damage mitochondria by the same mechanism.^(16,22)^ QD likely protects mitochondria by reducing ROS and maintaining oxidative phosphorylation. Mitochondrial ROS induce apoptosis by activating caspase-3 and caspase-9.^(22)^ For example, gastric acid activates caspase-3 via producing ROS, and subsequently induces apoptosis.^(27)^ In this study, we investigated apoptosis derived from mitochondria by studying the expression of caspase-9. In fact, treatment of BP accelerated activation of caspase-9. However, treatment of QD suppressed the activation of caspase-9 by BP (Supplemental Fig. 2*). QD would prevent BP-induced cell death by protecting mitochondria.

As mentioned above, QD is a mixture of indole compounds such as indigo and indirubin. It was reported that indole compounds have the ability to scavenge ROS.^(28)^ Furthermore, it is suggested that indole compounds bind to and activate the aryl hydrocarbon receptor (AhR).^(29)^ A previous study reported that QD improved colitis by increasing production of anti-inflammatory cytokines such as IL-10 and Il-22.^(7)^ Taken together, QD activates AhR and subsequently induces IL-22 via innate lymphoid cells.^(7,30)^ In addition, Hasnain *et al.*^(31)^ showed that IL-22 inhibited ROS via upregulating antioxidant genes *Gpx5* (encodes glutathione peroxidase-5), *Prdx5* (encodes peroxiredoxin-5), and *Sod-2* [encodes superoxide dismutase-2 (SOD2, also known as MnSOD)]. Therefore, QD is thought to scavenge ROS by promoting antioxidative gene expression.

However, indigo and indirubin alone didn’t prevent BP-induced cellular injury and death completely although, both indigo and indirubin have been reported QD’s main substance of O_2_^•−^ scavenger.^(1,2,8)^ When scavenging superoxide anions, it is necessary for the concentration of indigo and indirubin to be greater than 30 µM, but in this study, the concentrations were estimated to be 26.7 nM and 15.3 nM, respectively.^(8)^ We investigated the cellular protective ability of Indirubin and Indigo against BP. However, 10,20 µg/ml Indirubin and Indigo, which the concentrations are bigger than 30 µM, didn’t inhibit BP-induced cellular injury (Supplemental Fig. 3*). Furthermore, we tested the O_2_^•−^ scavenging ability of QD, indirubin and indigo by using Hypoxanthine/Xanthine oxidase (HX/XO) O_2_^•−^ production system in cell-free condition. Although 5 µg/ml and 30 µM of Indirubin and Indigo have an ability to scavenge O_2_^•−^, the effect was very slight (Supplemental Fig. 4*). However, the same concentration of QD (5 and 7.87 µg/ml) scavenged O_2_^•−^ effectively. Thus, Indirubin and Indigo, components of QD, may interact with other components to scavenge O_2_^•−^. Indeed, previous studies reported that QD has many unknown components.^(1,32)^ This study indicated that third components in QD have strong ability to scavenge ROS and contributed to protect cells.

We hypothesize that BP induces inhibition of oxidative phosphorylation and this effect is prevented or improved by QD. A previous study showed that prostaglandin B1 (PGBx) recovered phosphorylating ability and net adenosine triphosphate synthesis within mitochondria.^(33)^ It is surmised that QD, like PGBx, has the ability to accelerate mitochondrial oxidative phosphorylation.

In conclusion, QD scavenged ROS to prevent BP-induced gastric cellular injuries. We are currently performing *in vivo* studies to determine the detailed mechanisms underlying this process.
