# Supplementary material for: The preventive effect of Qing Dai on bisphosphonate-induced gastric cellular injuries
Source: J Clin Biochem Nutr. 2018 Nov 15;64(1):45–51. doi: 10.3164/jcbn.17-108 (PMC6348412; doi:10.3164/jcbn.17-108)
Supplement: Supplemental Figure 1 [file jcbn17-108sf01.pdf]

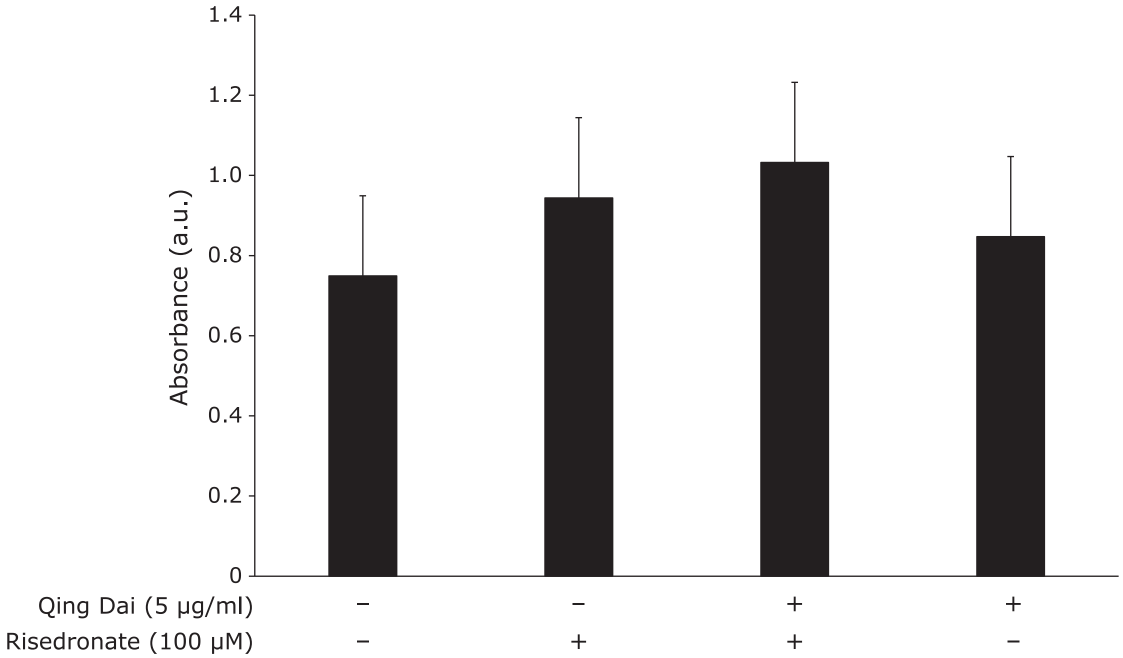

Supplemental Fig. 1. Ability of QD to inhibit uptake of BP was calculated by examining absorbance of cells (262 nm). Data are expressed as absorbance of cells (mean ± SD). *n* = 4.
