# Supplementary material for: The preventive effect of Qing Dai on bisphosphonate-induced gastric cellular injuries
Source: J Clin Biochem Nutr. 2018 Nov 15;64(1):45–51. doi: 10.3164/jcbn.17-108 (PMC6348412; doi:10.3164/jcbn.17-108)
Supplement: Supplemental Figure 2 [file jcbn17-108sf02.pdf]

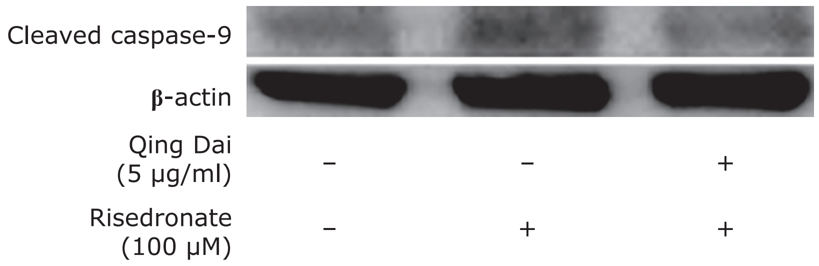

Supplemental Fig. 2. Western blotting analysis of caspase-9 was carried out using protein extraction fraction from RGM-1 cells which were treated in DMSO-containing medium or 100 μM risedronate-containing medium or 100 μM risedronate-containing medium with pretreatment of 5 μg/ml QD for 2.5 h. Cells were incubated for 24 h in each medium then cell lysates were prepared.
