# Supplementary material for: The preventive effect of Qing Dai on bisphosphonate-induced gastric cellular injuries
Source: J Clin Biochem Nutr. 2018 Nov 15;64(1):45–51. doi: 10.3164/jcbn.17-108 (PMC6348412; doi:10.3164/jcbn.17-108)
Supplement: Supplemental Figure 3 [file jcbn17-108sf03.pdf]

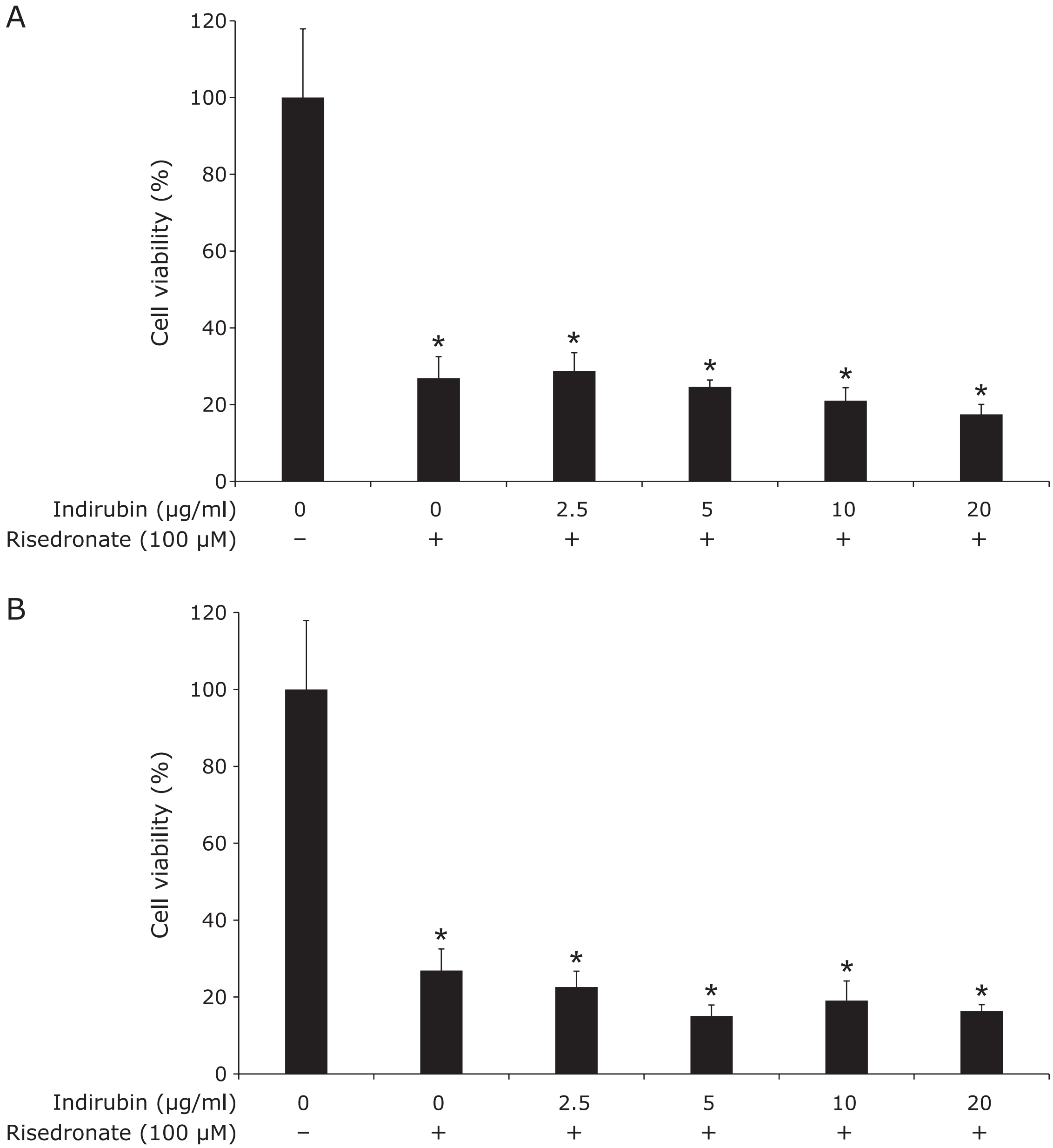

Supplemental Fig. 3. Cell viability was calculated using CCK-8 colorimetric assay. The prevention amount of Indirubin (A) and Indigo (B) for risedronate-induced cell injury was estimated. Data are expressed as percentages of untreated cells (mean ± SD). $n = 4$, *p<0.05, Tukey's test.
