# Supplementary material for: The preventive effect of Qing Dai on bisphosphonate-induced gastric cellular injuries
Source: J Clin Biochem Nutr. 2018 Nov 15;64(1):45–51. doi: 10.3164/jcbn.17-108 (PMC6348412; doi:10.3164/jcbn.17-108)
Supplement: Supplemental Figure 4 [file jcbn17-108sf04.pdf]

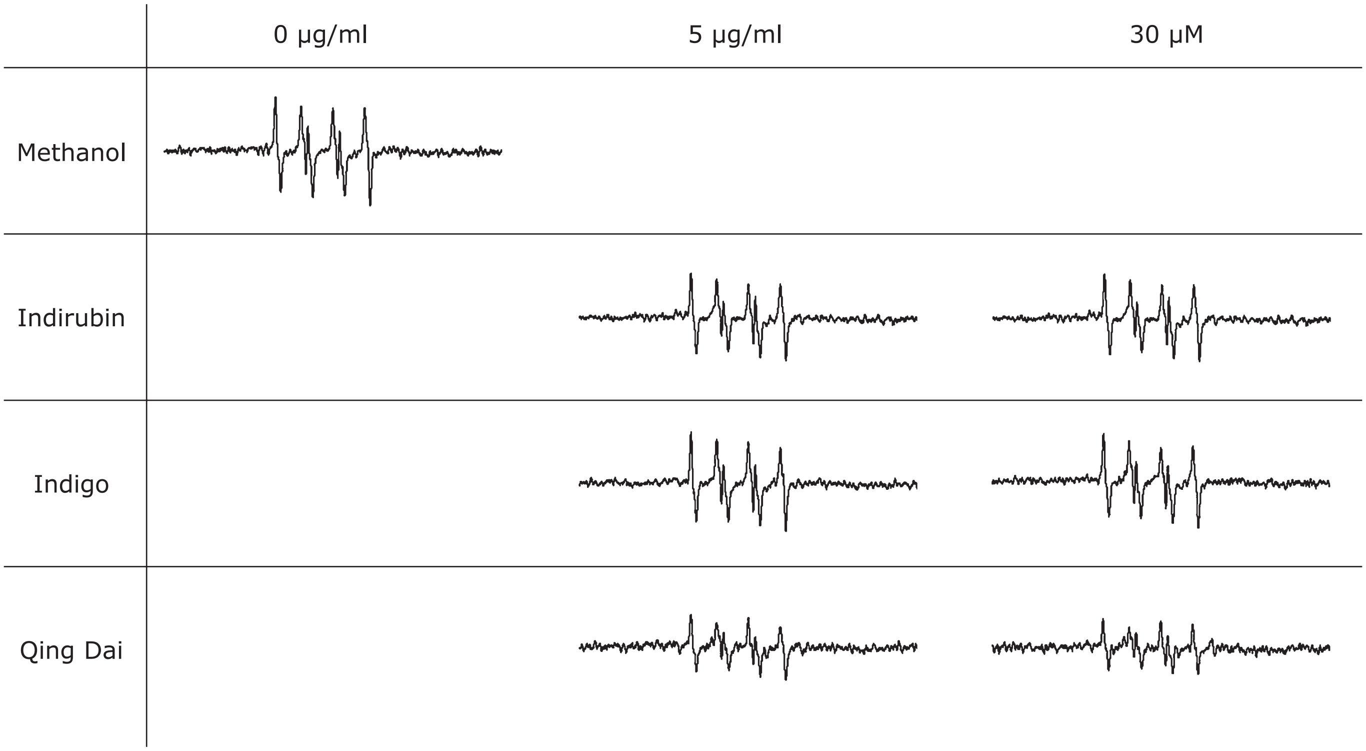

Supplemental Fig. 4. Hypoxanthine/Xanthine oxidase (HX/XO)-induced ROS were measured by ESR under cell-free condition. Briefly, 2 mM hypoxanthine and 0.5 U/ml of xanthine oxidase were reacted in PBS or methanol. DMPO was used as spin-trapping agent. PBS or methanol which contains DMPO was measured as control sample. 7.87 µg/ml QD was used as 30 µM QD.
